# The effectiveness of cognitive behavioral therapy on the quality of life of patients with inflammatory bowel disease: multi-center design and study protocol (KL!C- study)

**DOI:** 10.1186/1471-244X-12-227

**Published:** 2012-12-14

**Authors:** Floor Bennebroek Evertsz’, Claudi LH Bockting, Pieter CF Stokkers, Chris Hinnen, Robbert Sanderman, Mirjam AG Sprangers

**Affiliations:** 1 Department of Medical Psychology, Academic Medical Center, Meibergdreef 15, 1105 AZ, Amsterdam, the Netherlands; 2 Department of Clinical Psychology, University of Groningen, Grote Kruisstraat 2/1, 9712 TS, Groningen, the Netherlands; 3 Department of Gastroenterology, Sint Lucas Andreas Hospital, Jan Tooropstraat 164, 1061 AE, Amsterdam, the Netherlands; 4 Health Psychology Section, Department of Health Sciences, University Medical Center Groningen, University of Groningen, Antonius Deusinglaan 1 9713 AV, Groningen, the Netherlands; 5 Department of Medical Psychology, Slotervaart Hospital, Louwesweg 6, 1066 EC, Amsterdam, the Netherlands

**Keywords:** Inflammatory Bowel Disease, Cognitive behavioral therapy, Quality of life, Anxiety, Depression

## Abstract

**Background:**

Inflammatory Bowel Disease (IBD) patients report poorer quality of life (QoL) and more anxiety and depressive symptoms than controls from the general population. Cognitive behavioral therapy (CBT) is effective for anxiety and depression, but questionable in case of co-morbidity with IBD. Therefore, an adapted new CBT specifically designed for IBD patients was developed. The objective of this study is to evaluate the effectiveness of adapted CBT on QoL.

**Methods/design:**

IBD patients with a poor level of mental QoL (score less than or equal to 23 on the mental health scale of SF-36) will be randomly assigned to the experimental (n = 40) or waiting-list control condition (n = 40). The experimental condition will then immediately start CBT. The waiting-list control condition will wait 3,5 months before CBT begins with pre- and post assessments. Both conditions will complete a baseline and follow-up assessment following CBT and a mid-treatment assessment. The primary outcome is IBD-specific QoL (IBDQ). Secondary outcomes are generic QoL (SF-36) and anxiety and depression complaints (HADS, CES-D). Additionally, we will examine the working mechanism of the psychological intervention by investigating the impact of the intervention on illness-related cognitions, attitudes, coping styles and their associations with outcome. Data will be analysed on an intention to treat (ITT) as well as treatment completer basis (greater than or equal to five sessions followed).

**Discussion:**

If found effective, this IBD-specific CBT is a first step to enhance poor QoL in IBD patients and possibly, other gastroenterological diseases. By enhancing IBD patients’ QoL, we may also improve their mental and physical health, and lower unnecessary health care consumption.

**Trial registration number:**

NTR (TC = 1869)

## Background

According to the 2010 international Inflammatory Bowel Disease (IBD: Crohn’s disease and ulcerative colitis) task force meeting, incidence and prevalence of IBD is steadily increasing worldwide [1]. However, in high-incidence regions such as North America, the United Kingdom and Northern Europe the rates of IBD have stabilised [1,2]. In 2000, the incidence and prevalence of CD in North America were approximately 8 and 174 per 100,000, respectively and for UC approximately 8 and 214 per 100,000 respectively [3]. In Northern Europe these figures are slightly lower [2]. Approximately 50,000 people in the Netherlands have medically confirmed IBD. Each year this group increases with 2500 new patients [4]. IBD cannot be cured and the exact cause is unknown. The course of the disorder varies in severity but is characterized by unpredictability and is accompanied by physical symptoms such as diarrhoea, bowel spasms, faeces containing blood or mucus, qualms and fever attacks. Additionally, the medical treatments that, in some cases require life-long administration, often induce side effects and complications, such as weight gain, a moon face, eczema and acne. Peri-anal complications such as abscesses and fistulas severely affect quality of life by impairing sexual function and faecal continence and are a cause of chronic pain. Other manifestations such as spondylarthropathy may cause significant morbidity. Furthermore, many patients require surgical treatment in the course of their disease. Surgery frequently results in a permanent ileostoma or colostoma [5,6]. Moreover, chronic immune suppression that results from medical treatment increases the risk of hematological malignancies. Finally, since IBD is associated with an increased risk of colorectal cancer, patients undergo frequent endoscopic surveillance [7].

Consequently, the quality of life of IBD patients is impaired. Individuals with IBD have been found to report poorer quality of life and more anxiety and depressive symptoms than comparison-controls from the general population [8,9]. Many IBD patients are confronted with fatigue, pain, embarrassment and physical disablement that have negative effects on social and work life. Poor quality of life in turn, has a negative impact on relapse and disease activity [10].

Recent studies and clinical experience suggest that higher levels of anxiety and depressive symptoms in IBD patients are not only related to disease activity and severity. Individual characteristics also affect patients’ experienced distress, especially those associated with illness-related cognitions and attitudes [11]. Specific IBD-related cognitions (e.g. ‘I will never find a partner because of my colostomy bag’; ‘I can’t have sex because I am dirty’; ‘It makes no sense to invest in new work or personal relations because of the unpredictability of my illness’) and (premorbid) attitudes like perfectionism (‘Everything has to be perfect otherwise I am worthless’), dependency (‘Without the help of others I will not survive’) and mistrust (‘In the end other people will always let me down’) are highly influential in terms of ability to cope with IBD.

Of the psychotherapeutic intervention techniques that have been investigated, the most promising intervention for amending such dysfunctional cognitions and attitudes is cognitive behavioral therapy (CBT) [12]. This therapy posits that individual’s biased information processing leads to restrictive thoughts, feelings and behaviors that can culminate in anxiety and depressive symptoms and eventually in psychiatric disorders. CBT offers a well-developed intervention protocol that has been found to enhance quality of life and to decrease psychological distress. It has been found to be effective in people with other chronic illnesses such as chronic obstructive pulmonary disease, diabetes [13], and cancer [14].

A recent meta-analysis of 21 studies was conducted that evaluated the effect of psychological treatment on quality of life, emotional status and disease activity in IBD [15]. The psychological treatments included psycho-education as intervention (10 studies), stress management (3 studies), psychotherapy (6 studies) and other non-specific techniques like a support group (2 studies). Of all included studies only 6 studies evaluated the effect of a multi modular approach including IBD psycho-education (4 of them included CBT techniques). Overall, no significant effects of psychological interventions were found on improving health related quality of life, emotional problems and disease activity. All studies included suffered from various methodological shortcomings such as small sample sizes and non-random sampling. Moreover, all but one study included patients who did not have elevated levels of distress, thereby diluting the treatment effect. Patients in these studies seemed relatively healthy, so perhaps there was no room for improvement. Inclusion of patients with higher anxiety and depressive symptoms may have resulted in more positive effects. Only one study selected patients based on HADS anxiety levels [16]. Given these methodological shortcomings and the many multi-component interventions, it is still unclear what the effects of a specific CBT are. We have, therefore, developed a CBT that is specifically designed for patients with IBD who have a poor quality of life, thereby targeting a patient group that is most burdened by the illness.

We will examine the effectiveness of CBT in this study by using a randomized waiting-list control design, including a sufficiently large number of patients who have elevated levels of mental distress. This paper contains a detailed description of the design of this randomized controlled trial investigating the effectiveness of CBT specifically designed for IBD patients.

### Trial objectives and purpose

The primary objective is to investigate the effectiveness of individual CBT in a sample of IBD patients. Our primary outcome is the effect on IBD-specific quality of life (IBDQ). The secondary outcome is the effect of CBT on generic quality of life (SF-36), anxiety and depression complaints (HADS, CES-D) and illness activity (P-HBI and P-SCCAI). The secondary objective is to investigate the extent to which changes in quality of life and anxiety and depression are mediated by improvements in cognitions, attitudes and coping styles.

Our primary hypothesis states that cognitive behavioral therapy will improve quality of life, and reduce anxiety and depression. Our secondary hypothesis states that improvements in quality of life are mediated by changes in illness cognitions, attitudes and coping styles (avoidant and problem solving coping).

## Methods and design

**Figure 1:**
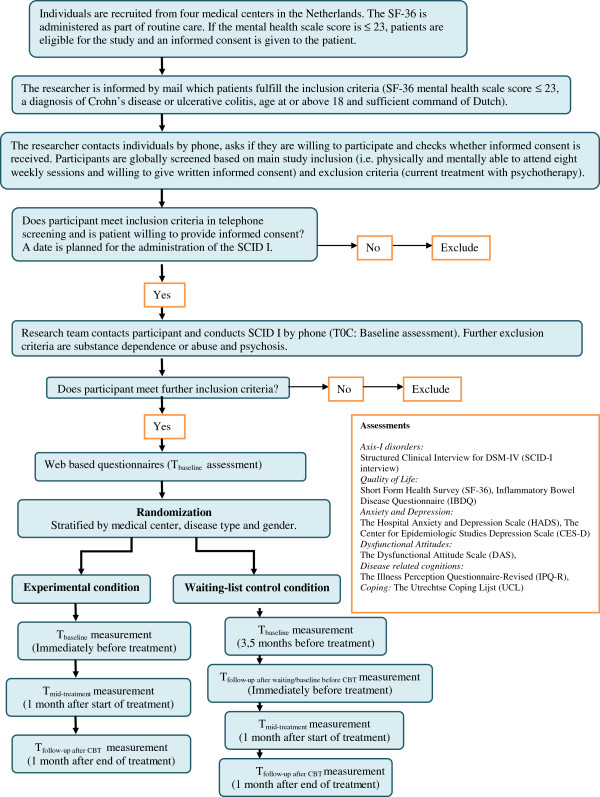
Flow chart study design.

The present study is a multi-center randomized clinical trial (see Figure 1). All patients with ulcerative colitis or Crohn’s disease attending the out-patient departments of the Academic Medical Center (AMC), VU University Amsterdam (VUmc), Flevo-hospital and Slotervaart-hospital will complete a 36-item questionnaire assessing generic health-related quality of life or health status (SF-36) prior to the consultation. This questionnaire will be administered as part of standard care. The nurse practitioner will be present and can be contacted for further information and help. Patients who score at or below the cut-off score of 23 on the mental health subscale will be eligible for the study [17]. If these patients are also 18 years or older, have a diagnosis of Crohn’s disease or ulcerative colitis and have sufficient command of Dutch, they will be given the informed consent letter by their treating gastroenterologist. After one week they will be contacted by telephone by the researcher to provide further information and/or clarification if needed. Also the researcher will check whether informed consent is received. If patients agree to participate, an appointment will be made to sign the informed consent letter in the presence of the researcher. Participants will be globally screened based on main study inclusion criteria (i.e. physically and mentally able to attend eight weekly sessions and willing to give written informed consent) and exclusion criteria (i.e. current treatment with psychotherapy). During this meeting an appointment will be scheduled for the administration of the Structural Clinical Interview for DSM-IV Disorders-I (SCID-I) that assesses Axis-I psychiatric diagnoses to determine further psychiatric exclusion criteria and to examine psychiatric disorders within this population (for instance anxiety and depression disorders) [18]. Patients will be further excluded from the study if they have current substance dependence or abuse, or psychosis.

The SCID-I will be administered by telephone, by psychologists who received a specific training for this.

All patients will then be asked to complete a set of questionnaires that assess the baseline primary and secondary outcomes via the web (see Table 1). If patients prefer paper and pencil completion, these questionnaires will be sent. After completion of the first baseline assessment, patients will be randomly assigned to the experimental or waiting-list control condition. Randomization will be stratified by gender, disease type and type of medical center (peripheral or academic) and will be performed by an independent employee at the Department of Clinical Epidemiology and Biostatistics of the AMC.

**Table 1:** Overview assessment schedule

**Measure**	**Description**	**T**_**baseline**_	**T**_**follow-up after waiting/baseline before CBT**_	**T**_**mid-treatment**_	**T**_**follow-up after CBT**_
*Screening SCID*-I^*^	DSM-IV-TR				
	Axis I disorders				
*Primary Outcome*					
IBDQ^**^	Inflammatory Bowel Disease Questionnaire	E C	C	E C	E C
*Secondary Outcome*					
SF-36^**^	MOS 36-item Short-Form Health Survey	E C	C	E C	E C
HADS^**^	Hospital Anxiety and Depression Scale	E C	C	E C	E C
CES-D^**^	The Center for Epidemiologic Studies Depression Scale	E C	C	E C	E C
P-HBI^**^	Patient-based Harvey Bradshaw Index	E C	C	E C	E C
P-SCCAI^**^	Patient-based Simple Clinical Colitis Activity Index	E C	C	E C	E C
*Potential Mediators*					
DAS^**^	Dysfunctional Attitude Scale	E C	C	E C	E C
IPQ-R^**^	Illness Perception Questionnaire-Revised	E C	C	E C	E C
UCL^**^	Utrechtse Coping Lijst	E C	C	E C	E C

*Experimental Condition* (*E*) *and Waiting list Control Condition* (*C*).

^*^Interview that is administered by telephone.

^**^Web-based questionnaire.

Participants assigned to the experimental condition will start treatment as soon as possible (maximum within 6 weeks). The second, mid-treatment assessment will take place four weeks following start of treatment and the third assessment one month following completion of treatment. Following the first baseline assessment, participants assigned to the waiting-list control condition will wait 3,5 months before they are treated with CBT. This period corresponds to the duration of the CBT intervention and follow-up assessment of the experimental group. After this 3,5 month waiting period the participants in the waiting-list control condition will be asked to complete a follow-up after waiting/baseline before CBT assessment before starting treatment. Analogous to the procedure in the experimental condition, the mid-treatment assessment will take place four weeks following start of treatment and the follow-up assessment one month following completion of treatment (see Table 1).

### Cognitive behavioral treatment manual

The treatment will consist of eight weekly sessions, each lasting one hour. The first session will focus on the rationale of cognitive behavioral therapy, i.e. the influence of (irrational or dysfunctional) cognitions and attitudes on (restrictive) feelings and behaviors. Additionally, goal setting will be initiated. Since patients may have a wide diversity of psychiatric problems (i.e. PTSD, anxiety disorders and depression), the treatment manual will encompass five optional modules for the therapist that focus on each of these disorders (i.e. exposure based for anxiety and PTSD, behavioral activation for depression). The subsequent sessions (2–6) will target teaching the patient to identify and challenge dysfunctional cognitions and attitudes related to IBD. Each session will address specific illness-related cognitions. If possible, dysfunctional cognitions and attitudes will be replaced by helpful cognitions and attitudes. Clearly, some of the dysfunctional cognitions are realistic and reflect the limitations that the disease imposes. Such thoughts will be worked through and intervention will focus on acceptance of these disease limitations. Finally, in the last two sessions, the newly learned cognitions and attitudes will be consolidated using specific CBT techniques such as flashcards with helpful cognitions or attitudes. As customary within CBT, each session patients will be given homework. The CBT will be conducted by registered psychotherapists, specialized in cognitive behavioral therapy.

All treatment sessions will be audio-recorded and therapists will receive feedback over the first two CBT treatments to promote treatment integrity. Additionally, regular supervision will be provided.

#### Module 1: identify and tackle avoidance behavior

During this module the patient will learn to identify his/her avoidance behavior in daily life that has an impact on anxiety and depressive symptoms. These symptoms lead to either an increase of anxiety and thereby an increase of avoidance behavior or to less interest while undertaking the activities and less satisfaction. This vicious circle and the importance of breaking this pattern will be explained to the patient. This will be done by identifying what specific situations are avoided by the patient in daily life and by eventually replacing avoidance behavior by alternative behavior. In case of depression the therapist will stimulate behavioral change by helping patients to become more active by increasing mood independent potential pleasurable (reinforcing) activities in their lives (behavior activation) [19-21]. The ultimate goal is to teach patients to actively react to difficult or painful situations or emotions instead of avoiding them (becoming pro-active rather than reactive to mood swings) [19].

#### Module 2: illness-history writing assignment

IBD patients often have a long illness history. This may consist of unprocessed negative experiences. For emotional processing of these negative experiences it is important that these experiences are identified and processed. Therefore, the patient will be asked to write about the most painful memories of his/her illness history in order to stimulate the processing of that memory [22,23]. The most painful memories are usually the ones that are being avoided or suppressed. The patient will write as detailed as possible about the most painful images (e.g. physician’s face, surgeon, hospital appliances, intensive care unit), thoughts (e.g. it is my fault, I am going to die) and emotions (e.g. embarrassment, feeling dirty and helpless). In a subsequent session the patient will read the most painful pieces of the assignment out loud. In this way the patient will be exposed to the most painful images, cognitions and emotions. Additionally, the therapist will identify the dysfunctional cognitions and attitudes of the patient.

#### Module 3: imagery with rescripting

The patient may experience intruding traumatic illness-related memories (flashbacks). During imagery with rescripting the patient will re-experience and rewrite the most painful memories in his/her imagination. Together the patient and therapist will compose a hierarchy of fearful situations and choose one or more of the most traumatic experiences to work through during imagery with rescripting. The patient will be asked to imagine the traumatic situation as lively as possible. After sufficient exposure the experience must be “re-written” following three phases. This will be done by returning to the negative experience in the patient’s imagination and altering it into satisfying new feelings, helpful attitudes and corrective experiences (e.g. support or presence of partner/parent) [24-26].

#### Module 4: cognitive therapy

In order to decrease emotions such as fear, sadness and anger cognitive therapy focuses on automatic negative thoughts and underlying dysfunctional attitudes that lead to these emotions. The cognitive model states that attitudes develop during childhood and are based on particular life events and/or views of significant others (e.g. parents or teachers) [12,27]. This determines one’s interpretation of one self, others and the world. For instance, an early experience such as being bullied at school can trigger the activation of dysfunctional attitudes such as ‘I am worthless’. Later in life, when a critical event, such as the diagnosis of IBD takes place, the dysfunctional attitudes may be activated. These, in turn, activate negative or anxious automatic thoughts such as ‘Now that I am sick, I am worthless’. These negative automatic thoughts can in turn cause anxiety and depressive symptoms. The ultimate aim of this module is to identify, challenge and alter dysfunctional attitudes of patients into helpful ones that fit in the current circumstances [28].

#### Module 5: relapse prevention plan

During the last two sessions the therapist will work with the patient on the steps that can be undertaken in order to prevent relapse. The patient will learn to signal potential relapse (e.g. symptoms like worrying, avoiding activities) and to develop prevention strategies. For instance, the patient might make use of tools such as flash cards with helpful attitudes, in order not to spiral back into the old dysfunctional ones [28].

### Sample size

We will consider a difference of 0.5 standard deviation, which is equal to a moderate effect size [29], clinically relevant. For calculating the required difference on the Inflammatory Bowel Disease Questionnaire (IBDQ), we will use the data from a study by McColl et al [30] among 111 IBD patients. The mean IBDQ score was 182 and the standard deviation was 59. A 0.5 standard deviation thus equals approximately 30 points on a scale ranging from 32 to 224. With an effect size of 0.5 standard deviation, an alpha level of 0.05, and a power of 80%, at least 34 patients in both the experimental condition and the waiting list control condition are needed. Given a potential attrition rate of 10%, we will include 40 patients in each condition.

#### Feasibility

Approximately 1600 IBD patients are treated at the AMC on a yearly basis. Given the fact that patients may be eligible regardless of time since diagnosis, the number of patients to be included in only one center is feasible. However, to enhance recruitment in a relatively short time period and promote future dissemination we will recruit patients in three additional medical centers (i.e. Slotervaart-hospital, Flevo-hospital and VUmc). Two of the four participating centers are academical medical centers (i.e. AMC and VUmc). Based on the literature [31], we expect 10-20% of the IBD patients to have a score below the cut-off point and be eligible for the study.

### Inclusion criteria and exclusion criteria

Inclusion criteria will be: (1) a diagnosis of Crohn’s disease or ulcerative colitis; (2) age above 18; (3) a score of 23 or lower on the mental health scale of the SF-36; (4) physically and mentally able to attend eight weekly sessions; and (5) sufficient command of Dutch. Exclusion criteria will be: (1) current psychological treatment; and (2) known psychiatric disorders that may complicate treatment (current substance dependence or abuse, current psychosis).

### Recruitment and informed consent

Patients who have a score of 23 or lower on the mental health subscale will be eligible for the study. The mental health subscale consists of 5 items that require a response on a 6-point scale (range 5–30). The cut-off score of 23 is chosen as scores of 23 or lower are found indicative (with a high level of sensitivity and specificity) of depression and anxiety in primary care patients [17]. If this step is ignored a failure of a positive effect may be contributed to high baseline scores and lack of room for further improvement [15]. Consequently, the researcher will contact patients by telephone to ask whether they are willing to participate and to check whether informed consent is received. If the participant meets all inclusion criteria and is willing to provide informed consent, an appointment will be made to administer the SCID-I by telephone.

The SCID-I will be used as a screening measurement before randomisation.

### Outcome measures

*Sociodemographic assessment* will include age, gender, educational level, employment status, marital status, ethnicity, and treatment center.

*Clinical physical data* will be completed by the treating gastroenterologist, and will include type of disease (Crohn’s disease and ulcerative colitis), date of onset of disease, having a colostomy, and number of operations. Finally, the biological markers of disease activity (e.g. C-reactive protein (CRP)) of each patient attending the outpatient department will be registered in an electronic database. Additionally disease activity will be measured using the following illness specific questionnaires: the Harvey Bradshaw index (P-HBI) for patients with Crohn’s disease and Simple Clinical Colitis Activity Index (P-SCCAI) for patients with ulcerative colitis. These patient-based questionnaires were specially developed for the current study [32]. The original SCCAI has good validity and reliability [33].

***Primary outcome measures ***the Inflammatory Bowel Disease Questionnaire (IBDQ) will be used to assess the primary outcome of the intervention [34]. The IBDQ measures health-related quality of life and consists of 32 items assessing four dimensions: bowel symptoms, systemic symptoms, emotional functioning, and social functioning. In addition to these four subscale scores, a total score can be calculated [20]. The IBDQ has good construct and criterion validity, test-retest reliability and high internal consistency [35].

***Secondary outcome measures ***the MOS SF-36 is a 36-item questionnaire assessing generic health-related quality of life or health status [36]. The items can be aggregated into a Physical Component Summary score and a Mental Component Summary score. The SF-36 has high internal consistency and good reliability and discriminant validity [37].

The Hospital Anxiety and Depression Scale (HADS) assesses the possible presence of anxiety and depressive states [38,39]. The HADS is considered to be unbiased by the presence of somatic illness and is found to be reliable and valid [40]. It consists of two sub-scales, anxiety and depression, both containing seven items. The CES-D scale (The Center for Epidemiologic Studies Depression Scale) is a short self-report scale designed to measure depressive symptomatology in the general population. The CES-D has high internal consistency, satisfactory test-retest reliability and excellent concurrent validity [41].

***Mediation variables ***The Illness Perception Questionnaire-Revised (IPQ-R) assesses illness-related cognitions. The 48 items measure seven dimensions: timeline acute/chronic, timeline cyclical, consequences, personal control, treatment control, illness coherence and emotional representations. The IPQ-R has good internal and test-retest reliability and predictive and discriminant validity [42].

The 40-item Dysfunctional Attitude Scale (DAS) measures dysfunctional attitudes, including excessive and rigid beliefs [43]. The DAS has good psychometric properties regarding reliability, internal consistency and convergent construct validity [44].

The Utrechtse Coping Lijst (UCL) assesses general coping behavior in problem situations [45,46]. The subscales ‘problem-solving’ (7 items) and ‘avoidance’ (8 items) will be used. The UCL has good test-retest reliability, construct and predictive validity [47].

All standard self-report questionnaires fulfill the following selection criteria. They: (a) are relatively brief, (b) have sufficient breadth of coverage, (c) are widely used, and (d) yield adequate to high levels of reliability and validity.

An overview of the questionnaires at the different time points for the experimental and waiting-list control conditions is provided (see Table 1).

### Withdrawal

Subjects can withdraw from the study at any time for any reason if they wish to do so without any consequences. Nevertheless, we will ask those who withdraw from CBT treatment if they are willing to complete the web-based questionnaires at the respective time intervals.

#### Safety monitoring and reporting

The study will be conducted according to the principles of the Declaration of Helsinki and in accordance with the Medical Research Involving Human Subjects Act (WMO).

The Medical Ethical Committee (MEC) of the AMC offers exemption from participants insurance.

In general there will be few if any risks associated with the research in question. Since there is no indication and no evidence that the intervention might be harmful to patients, we will not anticipate premature termination of the study. Clearly, each individual patient has the right to withdraw from the study at any time he/she wishes.

Suspected serious adverse events will be recorded and reported to the MEC of the AMC.

### Statistical analysis

Analyses of covariance will be conducted to determine the extent to which treated patients (experimental condition) show more improvement on the primary and secondary outcome variables compared to patients in the waiting-list control condition. A two-way factorial analysis of variance with the between-factor condition (experimental condition versus waiting list control condition) and the within-factor assessments will be used. Moreover, stratification variables, i.e. gender, treatment center and disease type will be included as covariates in the analyses (ANCOVA).

Additionally, the data of experimental and the waiting-list control condition will be pooled, to measure the overall effect of treatment. Before the two groups will be pooled, we will examine whether there is a difference in effect for the experimental versus the waiting-list control condition (Delta T_follow-up after CBT_-T_baseline before CBT_). In case differences emerge, this will be stratified reported.

For therapy effects, data will be analysed on an intention to treat (ITT) as well as treatment completer basis. The completers sample will include patients who attend at least 5 CBT sessions.

To express the magnitude of the effects, mean gain scores on the outcome measures will be standardized to Cohen’s *d*, representing the number of standard deviations separating the two means. Point estimates and 95% confidence intervals of *d* will be determined for the within and the between group effects. Between effect sizes will be calculated using the pooled standard deviation (of the baseline scores) and confidence intervals will be approximated from the central *t*-distribution.

#### Effect of CBT on quality of life mediated by cognitions, attitudes and coping

We will subsequently examine the extent to which changes in quality of life will be mediated by improvements in illness-cognitions, attitudes and coping styles and whether this change is associated with outcome.

## Discussion

Given its chronic nature and frequently reported poor quality of life for many patients, IBD is often associated with anxiety and depression. Apart from medical treatment, psychological intervention might be a crucial component of treatment of IBD patients and is needed alongside medical treatment [8,9]. Results from a recent meta-analysis of psychological interventions for treatment of IBD showed that current psychological treatment is not effective among IBD patients [15]. These disappointing results can, however, be attributed to specific treatment programs and a number of methodological short-comings of the evaluation studies. Therefore a further and more stringent study of psychological interventions in IBD patients to enhance quality of life and reduce anxiety and depression is warranted.

A number of strengths of the current study merit attention. First, it will offer a cognitive behavioral treatment manual, which is specifically tailored to the needs of IBD patients. Due to the focus on specific worries and needs of IBD patients, this treatment may be effective in enhancing quality of life and alleviating psychological symptoms, such as anxiety and depression associated with IBD. Additionally, this trial will allow us to investigate and learn more about the mechanisms of CBT. Particularly, illness-related cognitions, dysfunctional attitudes and coping behaviors (i.e. problem solving and avoidance) will be examined in order to understand the most effective components of CBT.

Second, only patients with a poor level of mental quality of life will be included. These patients are the ones who are most in need of psychological help. To the best of our knowledge, no study has previously selected patients based on their initial well-being. However, Larsson et. al. included their patients on another aspect of mental well-being, i.e. anxiety and depressive symptoms (patients with a high HADS anxiety levels) [16]. In this study an education program was employed instead of CBT, potentially explaining the lack of effect in the preselected patient group.

A third strength of the current study will be the random assignment of patients to the experimental group and the waiting list control group. To ensure an equal distribution among these two groups patients will be stratified by gender, treatment center and disease type. These factors are selected for several reasons. First, previous research found that disease course may differ for female and male patients. Particularly, females possibly have a higher risk of disease activity relapse than males [48]. Females, therefore, may have more psychological symptoms. Second, patients will be stratified by treatment center. A distinction will be made between an academic setting and a peripheral setting. We expect that an academic hospital would treat more severe cases of IBD than a peripheral setting. Finally, disease type is also used as a stratification factor. Previous research has found that CD patients undergo more surgical interventions and experience more disease exacerbations than UC patients [49]. This indicates that CD is a more complex disorder than UC. Additionally, CD patients report a poorer quality of life and more anxiety symptoms than UC patients [49,50]. Therefore, it is important that UC and CD patients are distributed evenly in the experimental group and the waiting list control group.

In sum, there is a compelling need to enhance the quality of life of patients with IBD, especially in patients with a low level quality of life. CBT may be the most promising intervention given its empirical status over 5 decades [12,27]. It is expected to enhance the quality of life of IBD patients. Poor quality of life is not only a consequence of the disease but may in itself be a causal factor. It may induce a higher level of symptom severity, more reporting of unexplained physical symptoms and poorer treatment adherence. If found feasible and effective, this illness-oriented CBT is a first step to enhance poor quality of life in patients with IBD and, possibly, other gastroenterological diseases.

### Ethical considerations

The current study protocol was approved by the MEC of the AMC Amsterdam and confirmed by the institutional ethics review committees from the participating medical centers (i.e. Flevo Hospital, Slotervaart Hospital, VUmc).

## Competing interests

The authors declare that they have no competing interests. F. Bennebroek Evertsz’ received an unrestricted research grant from Scheringh and Plough of 20.000 euros to study psychological factors in IBD.

## Authors’ contributions

FBE drafted this paper (which was added and modified by all other authors), wrote the treatment manual for the used CBT intervention and was responsible for the training and supervision of the psychotherapists. MAGS is the study’s principal investigator. All authors were responsible for the funding, contributed to the design of the study and to the analytic strategy. All authors read and approved the final manuscript.

## Pre-publication history

The pre-publication history for this paper can be accessed here:

http://www.biomedcentral.com/1471-244X/12/227/prepub
